# Fused ensembles of dynamic-rupture earthquake simulations to accelerate Bayesian inference

**DOI:** 10.1007/s13137-026-00287-6

**Published:** 2026-04-09

**Authors:** Vikas Kurapati, David Schneller, Linus Seelinger, Zihua Niu, Alice-Agnes Gabriel, Michael Bader

**Affiliations:** 1 School of Computation, Information and Technology, Technical University of Munich, Boltzmannstr. 3, Garching, 85748 Germany; 2 Scientific Computing Center, Karlsruhe Institute of Technology, Kaiserstraße 12, Karlsruhe, 76131 Germany; 3 Department of Earth and Environmental Sciences Geophysics, Ludwig-Maximilians-Universität, Theresienstr. 41, Munich, 80333 Germany; 4 Scripps Institution of Oceanography, University of California, San Diego, 9500 Gilman Dr #0225, La Jolla, CA United States of America

**Keywords:** Uncertainty quantification, Fused simulations, Earthquake simulation, Bayesian inference, Dynamic rupture, Discontinuous Galerkin

## Abstract

Understanding earthquake dynamics is essential for seismic hazard assessment and risk mitigation. In this context, Bayesian inference provides valuable insights into model parameters by combining simulation models with real-world data. Such Bayesian parameter inference with uncertainty quantification (UQ) requires numerous simulation runs and is therefore often computationally infeasible. Already, a single high-fidelity earthquake simulation – governed by a linear hyperbolic seismic wave equation coupled nonlinearly to a friction law and plastic deformation – is computationally expensive. In this study, we investigate the use of fused ensemble simulations to accelerate large earthquake simulation workflows and UQ studies. We implement and evaluate this approach in SeisSol, a high-performance computing software for the simulation of complex earthquake events that uses an Arbitrary high-order DERivative Discontinuous Galerkin (ADER-DG) scheme. Via fused ensembles, we turn the element-local small sparse/dense matrix operations into tensor contractions working on a dense rank-3 tensor and sparse matrices. These are again executed via loops of small, sparse/dense matrix operations, but with better computational efficiency, due to better exploitation of SIMD instructions on CPUs. We also compare two implementation variants (with different implementation effort) for kernels modeling non-linear dynamic rupture and effects of material plasticity. Our results demonstrate that fused simulations can be up to 5.54 times faster than a single execution – though this depends strongly on the discretization order, the problem size, and the compute architecture. For a full UQ example workflow, we demonstrate a speedup of 1.6, resulting in 36% savings in node hours for the entire workflow.

## Introduction

In computational seismology, many approaches rely on performing large ensembles of earthquake simulations. This includes approaches to physics-based seismic hazard assessment (Callaghan et al. 2024; Cui et al. 2013; Mert et al. 2016, e.g.), uncertainty quantification and Bayesian inference of modeling parameters (Premus et al. 2022; Schliwa et al. 2024, e.g.) or even rapid-response simulations (Zuccolo et al. 2025). Such *ensemble simulations* share the need for performing a vast number of simulations with small variations in the input parameters (such as frictional parameters governing dynamic rupture or plastic deformation) or underlying physical conditions (subsurface stress state, source characteristics, e.g.). As models for the physical processes governing earthquake rupture and seismic wave propagation are getting more and more complex, the computational effort for accurately simulating earthquake phenomena in realistic three-dimensional Earth models is growing, as well. A single high-resolution earthquake simulation can easily require tens to hundreds, or even thousands of compute nodes of a supercomputer over several hours (e.g., Akcelik et al. 2003; Cui et al. 2010; Heinecke et al. 2014).

The high computational cost of running large ensembles provides motivation to exploit all avenues for reducing both the invested compute hours and the time to completion of such studies. Efficient parallelization of UQ algorithms and of the simulation runs typically aims at the latter: while the most cost-efficient (in terms of invested compute hours) way to execute a UQ workflow might be to execute all simulations sequentially and on the smallest-possible number of compute nodes – to not lose any performance due to lack of scalability – keeping the time to completion of such a workflow within acceptable limits, requires parallelization of both, accepting a certain imperfection in parallel efficiency. On the other hand, using highly sophisticated UQ algorithms that reduce the number of simulation runs as much as possible, or optimizing the time-to-solution of the individual simulation runs, will attempt to reduce the invested compute hours for the ensemble simulations.

In this work, we explore the potential of so-called *fused ensemble simulations* (Breuer et al. 2017; Parno et al. 2021; Uphoff and Bader 2020, e.g.) as an additional avenue to accelerate large-scale UQ workflows. We extend the earthquake simulation software SeisSol (www.seissol.org) (Gabriel et al. 2025) to support the execution of several earthquake model evaluations with dynamic rupture and off-fault plasticity into one single *fused* simulation run. This is achieved by adding an additional dimension to all degree-of-freedom tensors. In performance-dominant wave propagation kernels, this leads to the generation of more efficient sparse and dense element-local matrix operations by the code-generation infrastructure – better exploiting vector operations, improving alignment with cache lines, and avoiding zero-padding for non-aligned compute loads. We discuss two variants to illustrate implementation trade-offs for additional physics kernels that cannot be entirely covered by code generation.

We enable the integration of fused simulations into general, potentially complex UQ workflows using the ‘Uncertainty Quantification and Model Bridge’ (*UM-Bridge*, https://github.com/um-bridge) (Seelinger et al. 2025), which provides a flexible interface between UQ algorithms and simulators. Specifically, we introduce a reusable component that batches UQ model evaluation requests for fused execution. Fused simulations can thus be triggered for any UQ algorithm that offers parallelism, and for any simulation code that profits from launching fused ensembles.

We evaluate the performance gains due to fused simulations for a dynamic rupture community benchmark (Harris et al. 2018) problem (TPV13^1^), and for two production scenarios: simulation of the 2019 Searles Valley $\hbox {M}_w$ 6.4 Earthquake (Taufiqurrahman et al. 2023) and of the 2023 Kahramanmaraş, Turkey, $\hbox {M}_w$ 7.8–7.7 Earthquake doublet (Gabriel et al. 2023; Jia et al. 2023). We compare results on three CPU-based supercomputers (Vista, Frontera, and SuperMUC-NG), featuring Arm and Intel CPUs, and identify hardware-specific differences in the efficiency of fused simulations, e.g. due to the different width of SIMD registers. Finally, we showcase the potential to increase the number of simulations performed per available compute resources for UQ workflows on a Bayesian inference scenario (Kruse et al. 2024; Niu 2025), to determine the posterior distribution of a depth-dependent rate-and-state friction parameter (Dunham et al. 2011) of the dynamic rupture process modeling the 2019 $\hbox {M}_w$ 6.4 Searles Valley Earthquake.

In Section 2, we outline the aspired workflows for Bayesian inference of parameters for large-scale physics-based earthquake simulations. We provide an overview of related work in Section 3. In Section 4 and Section 5, we summarize the high-order discontinuous Galerkin discretization used in SeisSol, introduce our fusion strategy, and discuss the challenges of applying it to non-linear physics models. In Section 6, we describe the software architecture to smoothly integrate fused simulations into *any* parallel UQ workflow, by extending UM-Bridge with a component to transparently batch simulation requests. Section 7 is dedicated to performance and scaling results for the benchmark and production scenarios, and Section 8 presents results for exploiting fused simulations in an example workflow with UM-Bridge and SeisSol. Finally, we conclude and point out potential directions for future research in Section 9.

## Background: Bayesian inference of parameters for large-scale physics-based earthquake simulations

While fused ensembles can be considered for any simulation workflow that involves potentially parallel or concurrent execution of many simulation tasks (see Section 3 for related work), our study focuses on a concrete setup: we consider *Bayesian inference* workflows to determine the probability distribution of physical parameters from observational data. In particular, we build on previous work (Kruse et al. 2024) on parallelization of the Multi-Level Delayed Acceptance (MLDA) algorithm (Lykkegaard et al. 2023), which examines the impact of three parallelization routes – parallel chains, additional prefetching (see overview below) and multi-node execution of the simulations themselves – on the overall performance of the UQ workflow. Respective workflows are designed for Bayesian inversion of parameters of complex 3D earthquake simulations, such as parallel work by Niu (2025), which resolves on- and off-fault parameters for dynamic rupture earthquake simulations with off-fault plasticity, using high-resolution satellite imagery and global navigation-system data.

We use SeisSol (Gabriel et al. 2025) to simulate complex earthquake dynamics. In this work, we rely on the following modeling features:

- seismic wave propagation in elastic materials (Dumbser et al. 2007) with off-fault inelastic deformation (Wollherr et al. 2018) (when stresses exceed the limit for elastic response), implemented using a visco-elasto-plastic Drucker-Prager rheology (Andrews 2005);
- dynamic rupture earthquake sources (Pelties et al. 2012) based on various friction laws like linear slip weakening (Ida 1972; Palmer et al. 1591), or rate and state friction laws (Dieterich 1979; Ruina 1983);
- clustered local time stepping (LTS) to use different time-steps in the computational domain with the ADER-DG method (Breuer et al. 2016; Uphoff et al. 2004).

SeisSol uses high-order discontinuous Galerkin discretization with arbitrary high-order derivative time-stepping (ADER-DG) (Dumbser et al. 2007) on unstructured adaptive tetrahedral meshes – see Section 4.1 for details.

In our target UQ workflows (Kruse et al. 2024; Niu 2025), we aim to determine the physical parameters governing on-fault friction in the dynamic rupture process and off-fault inelastic deformation due to strong dynamic stress variations, and how these can be inferred from surface displacement data. We quantify uncertainties and correlations among the physical parameters using sampling-based Bayesian inversion methods. In Kruse et al. (2024), we introduced prefetching simulation runs as a way of parallelization in Multilevel Delayed Acceptance (MLDA) (Lykkegaard et al. 2023). MLDA is closely related to the earlier Multilevel MCMC approach of Dodwell et al. (2015) and to delayed-acceptance MCMC (Christen and Fox 2005). Like its spiritual ancestor Multilevel MCMC (Dodwell et al. 2015), MLDA exploits a hierarchy of models with increasing fidelity and respective cost. Combining a few evaluations of the compute-intensive fully-resolved model with many evaluations of fast approximate models can significantly reduce compute cost while achieving accurate inversion results. In Kruse et al. (2024), different convergence orders in ADER-DG (3 vs. 4) are chosen as levels of model accuracy, and approximately twice as many models of convergence order 3 are evaluated as at the finest level of convergence order 4.

We employ the UM-Bridge interface (Seelinger et al. 2025) in order to seamlessly integrate our UQ software with SeisSol running on a high-performance computing (HPC) cluster. In addition to acting as a language-agnostic bridge between simulators and higher-level analysis tools, UM-Bridge handles parallel simulation jobs on HPC systems in a way that is entirely transparent to UQ software. Thanks to this universal interface, any higher-level analysis workflow (including UQ, machine learning, and optimization) can now benefit from batched simulations if the forward simulator supports them.

***Assumptions and Potential:***

In the following, we will combine potentially concurrent simulations in the workflow into *fused ensembles*. To efficiently implement such ensembles, we assume that all fused simulations work on a “similar” setup – more concretely, we demand that:

1. The discretized mesh is identical for all simulations, and all simulations have the same order of convergence. This ensures that all element updates (incl. neighbor contribution, etc.) follow the same numerical scheme for all fused simulations.
2. While this enforces a uniform fault geometry for all fused simulations, fault properties such as parameters for static or dynamic friction, etc., can be varied.
3. All the simulations share the same material parameters. This implies that all simulations can run at the same (local) time step sizes, which are constrained by material parameters and element sizes.
4. The simulated time of all fused simulations, as well as any output settings (frequency of output, position of receivers, etc.), is identical.

We expect that these limitations are typically fulfilled by many simulation workflows for various applications, particularly to model uncertainties, estimate parameter distributions, or computing inverse problems, where algorithms such as Monte-Carlo-type sampling and Bayesian inference require numerous simulation runs that do not change the simulation setup significantly.

To the best of our knowledge, the explicit strategy of fusing multiple simulations into a single execution of a complex, high-fidelity solver such as SeisSol has not been extensively coupled with UQ frameworks. Our work fills this gap by introducing and analyzing fused ensembles to improve throughput and scalability in large-scale Bayesian inference tasks involving earthquake simulations.

## Related work

***Fused simulations:***

Breuer et al. (2017) investigated the possibility of fusing multiple simulations for seismic wave propagation, using the same ADER-DG method as SeisSol with an isotropic elastic model, reporting a speedup of 2.1 for a fourth-order scheme when fusing eight simulations in a single run compared to eight individual simulations. We follow a similar approach for wave propagation, but consider a full earthquake simulation with dynamic rupture and plasticity, exploiting SeisSol’s code-generation approach based on the YATeTo package (Uphoff and Bader 2020) for small tensor operations.

Fused solution of problems is more commonly utilized in the context of solving large (typically sparse) systems of equations with multiple right-hand sides. For example, early work by Feng et al. (1995) introduced a block conjugate gradient method to solve symmetric and positive definite linear systems simultaneously with multiple right-hand sides, addressing requirements of non-linear structural analysis problems, where multiple right-hand sides correspond to multiple loading scenarios. Liu et al. (2012) presented an algorithmic approach to redesign sparse matrix-vector products within iterative solvers, introducing a generalized sparse-matrix-vector (SPMV) kernel that computes the matrix product with multiple vectors simultaneously. Many respective approaches are oriented towards specific applications and/or specific compute architectures. Walden et al. (2016) uses fused multiple right-hand sides for a specific kernel in lattice quantum chromodynamics and optimizes for the Intel Knights Corner manycore architecture. Imamura et al. (2016) study performance of iterative solvers for multiple right-hand-side vectors for exascale candidate architecture. Richtmann et al. (2016) processes multiple right-hand sides simultaneously to better amortize the expensive setup step of the DD-$\alpha$ algebraic multigrid method. Bastian et al. (2016) implements fused ensembles in the Finite Element framework DUNE, to enable solving linear systems with multiple right-hand sides for general elliptic and parabolic PDE solvers. Kühn et al. (2023) address SIMD vectorization on CPU architectures, introducing a specific SIMD data type in their Sparse Linear System Solver library (Spliss) for solving locally varying linear systems with multiple right-hand sides.

In computational fluid dynamics, ensemble averaging is used to retrieve statistics of turbulence quantities, e.g. In that context, Makarashvili et al. (2017) analyzes the potential performance benefits of using ensemble simulations to calculate statistical quantities in large eddy simulations. Gunzburger et al. (2018) uses the same technique to accelerate fused ensembles with different viscosities. Krasnopolsky (2018) considers incompressible turbulent flow simulations (using direct numerical simulation, DNS) based on optimizing solvers for multiple right-hand sides. Mohebujjaman and Rebholz (2017) proposed an algorithm for computing flow ensembles of incompressible magneto-hydrodynamic flows under uncertainties in initial or boundary data by exploiting block linear solvers.

Phipps et al. (2017) envisage UQ workflows, in particular, realizing multiple-right-hand-side solvers for elliptical problems in the Trilinos package (Heroux et al. 2005), based on the performance portability framework Kokkos (Edwards et al. 2014).

***Earthquake simulation workflows:***

Modern earthquake simulation frameworks integrate earthquake source characteristics, seismic wave propagation, and hazard analysis on HPC platforms. For example, the Statewide California Earthquake Center (SCEC)’s Cybershake system computes physics-based seismic hazard models by simulating kinematic finite-fault earthquake scenarios and 3D seismic wavefield simulations within a unified HPC workflow (Rojas et al. 2024; Callaghan et al. 2024). High-order codes like SeisSol and SW4 are leveraged to model seismic wave propagation in fully 3D heterogeneous domains on supercomputers (Taufiqurrahman et al. 2022; Petersson et al. 2017). These workflows employ automation to orchestrate thousands of parallel runs; for example, a recent CyberShake campaign executed around 32,000 jobs with 2.5 PB of output on the Summit supercomputer (Callaghan et al. 2024). Prototype rapid-response systems like UrgentShake also leverage HPC to generate physics-based ground motion scenarios in near real time (Zuccolo et al. 2025). Such integrated HPC-enabled workflows thus link detailed rupture modeling, full-wave propagation, and hazard outputs in a single workflow.

## Physics-based earthquake simulations using SeisSol

In this section, we outline the numerical algorithms in SeisSol, as far as they are required to understand the implementation and performance impact of fused simulations. We focus on the ADER-DG scheme for wave propagation (Section 4.1), the dynamic rupture process (Section 4.2), and treatment of off-fault plasticity (Section 4.3).

### The ADER-DG scheme for elastic wave propagation

We consider the elastic wave equation in first-order form (using Einstein convention)

1 $$\begin{aligned} \frac{\partial q_i}{\partial t} + A_{ij} \frac{\partial q_j}{\partial x} + B_{ij} \frac{\partial q_j}{\partial y} + C_{ij} \frac{\partial q_j}{\partial z} = 0, \end{aligned}$$

where $\textbf{q}$ contains the space-time dependent state variables

2 $$\begin{aligned} \textbf{q} = \left( q_1, \dots , q_9 \right) ^T := \left( \sigma _{11}, \sigma _{22}, \sigma _{33}, \sigma _{12}, \sigma _{23}, \sigma _{13}, v_1, v_2, v_3\right) ^T, \end{aligned}$$

in which $\sigma _{ij}$ are the components of the symmetric stress tensor and $v_1, v_2, v_3$ are particle velocities in *x*, *y*, *z* directions. The space-dependent linear operators $\textbf{A}, \textbf{B}, \textbf{C}$^2^ govern the linear PDE system, and depend on the local material properties (Dumbser and Käser 2006). SeisSol solves the PDE system (1) on an unstructured tetrahedral mesh, using high-order discontinuous Galerkin discretization with Arbitrary high-order Derivatives (ADER) time-stepping (Titarev and Toro 2002). The resulting ADER-DG scheme combines a high-order element-local predictor with a corrector step that considers how element-local solutions depend on neighbor elements by solving the Riemann problem at the element faces. We refer to Käser and Dumbser (2006); Dumbser and Käser (2006); Dumbser et al. (2007) for details of the ADER-DG method and to Uphoff et al. (2004); Uphoff and Bader (2020) for details of implementation in SeisSol.

For the context of this paper, we will focus on how the respective numerical steps are implemented in SeisSol. In each element *k*, the numerical solution of timestep *n* is stored as a matrix $\textbf{Q}_k^n$ of polynomial coefficients with the quantities and polynomial basis functions as row and column dimensions of the matrix, respectively. Choosing orthogonal polynomial basis functions in space, DG discretization of (1) yields the discretized derivative operator (omitting indices *k* and *n* for better readability)

3 $$\begin{aligned} D_{ij} := \hat{K}_{im}^\xi Q_{ml}A_{lj}^* + \hat{K}_{im}^\eta Q_{ml}B_{lj}^* + \hat{K}_{im}^\zeta Q_{ml}C_{lj}^*, \end{aligned}$$

where $\hat{\textbf{K}}^\xi = \textbf{M}^{-1} \left( \textbf{K}^\xi \right) ^T$ (similar for $\hat{\textbf{K}}^\eta ,\hat{\textbf{K}}^\zeta$), with stiffness matrices $\textbf{K}^\xi$, $\textbf{K}^\eta$ and $\textbf{K}^\zeta$ and diagonal mass matrix $\textbf{M}$. $\textbf{A}^*$, $\textbf{B}^*$ and $\textbf{C}^*$ are linear combinations (depending on the geometric orientation of the element) of the flux matrices $\textbf{A}$, $\textbf{B}$ and $\textbf{C}$ from (1).

Following the Cauchy–Kovalevskaya procedure, the first step in the ADER-DG scheme is now to compute an element-local space-time predictor solution

4 $$\begin{aligned} \textbf{L}(t + \tau ) = \textbf{Q}(t) + \tau \textbf{D}^1 + ... + \frac{\tau ^{P}}{P!}\textbf{D}^{P}, \end{aligned}$$

where *P* is the degree of the polynomial basic functions of the DG scheme, leading to a convergence order of $P+1$ (Cockburn et al. 2000). $\textbf{D}^p$ is an approximation of the *p*-th derivative. $\textbf{D}^1,...,\textbf{D}^{P}$ are computed by recursively applying (3):

5 $$\begin{aligned} D^{p + 1}_{ij} := \hat{K}_{im}^\xi D^p_{ml}A_{lj}^* + \hat{K}_{im}^\eta D^p_{ml}B_{lj}^* + \hat{K}_{im}^\zeta D^p_{ml}C_{lj}^* \qquad D^0_{ij} := Q_{ij}. \end{aligned}$$

Integrating eq. (4) over a time step $\Delta t$ yields the *predictor* values

6 $$\begin{aligned} {I}_{ij} = \sum _{p = 0}^{P} \frac{\Delta t^{p+1}}{\left( p + 1\right) !} {D}^p_{ij}, \end{aligned}$$

from which we compute the updated unknowns $Q_{ij}^{n+1, *}$ for timestep $n+1$ via a *local corrector* step. The latter consists of a cell-local part,

7 $$\begin{aligned} \begin{aligned} Q_{ij}^{n+1, *} =&\; Q_{ij}^{n} + \tilde{K}_{im}^\xi I_{ml}A_{lj}^* + \tilde{K}_{im}^\eta I_{ml}B_{lj}^* + \tilde{K}_{im}^\zeta I_{ml}C_{lj} ^* \\&- \frac{1}{\left| J\right| } M^{-1}_{il}\biggl (\sum _{f=1}^4\left| S_f\right| F^{-,f}_{lm}I_{mn}{A}^{*+}_{nj}\biggr ) \end{aligned} \end{aligned}$$

and time-integrated contributions $I^{f}_{mn}$ from the four neighbor elements $f=1, \dots , 4$, included via a *neighbor corrector*:

8 $$\begin{aligned} Q_{ij}^{n+1} = Q_{ij}^{n+1, *} - \frac{1}{\left| J\right| } \textbf{M}^{-1}_{il} \biggl (\sum _{f=1}^4 \left| S_f\right| {F}^{+,f}_{lm} I^{f}_{mn}{A}^{*-}_{nj}\biggr ). \end{aligned}$$

In eqs. (7) and (8), $\tilde{\textbf{K}}^\xi = \textbf{M}^{-1} \textbf{K}^\xi$ ($\eta$, $\zeta$ similar), $\left| J\right|$ is the volume of the element, and $\left| S_i\right|$ the surface area of the *i*-th face. The flux matrices $\textbf{F}^{-,i}$, $\textbf{F}^{+,i,j,h}$ for each face *i* depend on the choice of the basis function and the relative position of the element with the respective neighbor (Dumbser and Käser 2006). ${\mathbf {A^*}}_k^+$, ${\mathbf {A^*}}_{k\left( i\right) }^-$ are the matrices considering the solution of the elastic Riemann problem at the boundaries which contribute to the numerical fluxes.

The entire predictor-corrector scheme, as outlined in eqs. (5) to (8), can be expressed as element-local matrix and/or tensor operations and is implemented via many small matrix multiplications (or tensor contractions), as explained in Section 4.4.

### Dynamic rupture

SeisSol simulates the dynamic rupture process by modeling fault slip as an internal boundary condition coupled to wave propagation (Bizzarri and Cocco 2003; De La Puente et al. 2009; Pelties et al. 2012). First, the tractions (aligned to the fault plane, as resulting from the stresses) and velocities on both sides of the prescribed fault are computed. We then check if the fault remains locked or is sliding. Sliding causes a relative displacement across the fault interface (“slip”). The behavior of sliding and slip is governed by **nonlinear friction laws**, which relate the shear traction to slip rate: Traction $\textbf{t}$ is split into shear traction $\boldsymbol{\tau }$ and a normal component $\textbf{t}_\text {n}$, with $\textbf{t} = \boldsymbol{\tau } + \textbf{t}_\text {n}$. The fault slides, if the shear traction is larger than the fault strength

9 $$\begin{aligned} \tau _s = \max (0, -\mu _f t_\text {n} - C). \end{aligned}$$

*C* is a constant cohesion parameter which adds to the fault strength and $t_\text {n} = \textbf{t}_\text {n} \cdot \textbf{n}$ is the normal component of the traction. Note that, by convention, a negative $t_\text {n}$ denotes compression. Following (9), the fault strength $\tau _s$ is connected to the normal component $t_\text {n}$ of the traction via a parameter $\mu _f$, which is determined by a friction law

10 $$\begin{aligned} \mu _f = f(||\textbf{s}||, \psi ), \qquad \frac{\partial \psi }{\partial t} = g(||\textbf{s}||, \psi ), \end{aligned}$$

where the friction law functions *f* and *g* depend on a state variable $\psi$ and on the slip rate $\textbf{s} = \llbracket \textbf{v} \rrbracket$, which is the difference in velocities across the fault. The differential equation (10) and eq. (9) need to be solved iteratively to obtain the tractions and velocities across the fault that satisfy the partial differential equation.

Thus, the dynamic rupture implementation in SeisSol performs the following steps (in a loop over all dynamic rupture faces):

1. Compute the element-local space-time predictor solution $\textbf{L}\left( t + \tau \right)$ as in (4), but evaluate the 3D space solution at all $P+1$ intermediate time quadrature points.
2. Project the 3D space-time solution computed in step 1 onto the 2D dynamic rupture face, which gives the values of the stresses and velocities at each space-time quadrature point. This is done on both sides of the fault.
3. For each space time quadrature point on the face:
  - Evaluate an intermediate state by solving the Riemann problem (assuming elasticity), using the values of stresses and velocities on either side of the fault.
  - With the obtained intermediate state, check if the fault is locked or sliding by evaluating the fault strength as per (9). If the fault is locked, the intermediate state is taken as the final state for flux calculations.
  - If the fault is sliding, compute the slip rate iteratively from eqs. (9) and (10) and evaluate an imposed state for tractions and velocities which satisfy these conditions.
4. With the final tractions and velocities, calculate a correction flux to replace the respective neighbor fluxes in eqs. (7) and (8).

Note that while some of the steps (3D$\rightarrow$2D projection, Riemann solution) of the dynamic rupture implementation can again be implemented via small matrix or tensor operations, the nonlinear equations eqs. (9) and (10) have to be treated separately when fusing the simulations (cf. Section 5.2).

### Off-fault plasticity

Off-fault plastic deformation is implemented using a visco-elasto-plastic Drucker-Prager rheology (Andrews 2005; Dunham et al. 2011; Erickson et al. 2017; Templeton and Rice 2008; Xu et al. 2012). At each time step, we first compute the quantities $Q_{ij}^{n+1}$ via (8), i.e., assuming purely elastic behavior. To check whether the respective trial stresses $\sigma ^\text {trial}$ exceed elasticity limits and cause plastic deformation, we first need to transform the “elastic” solution $Q_{ij}^{n+1}$, which is represented via modal basis functions (orthogonal polynomials), into the corresponding nodal representation (based on Lagrange polynomials). We then evaluate at each nodal point a yield function $f_\text {yield}$ that determines whether plastic deformation occurs. If $f_\text {yield}\left( \sigma ^\text {trial}\right) < 0$, the material remains elastic at this point; otherwise, we trigger a modification of the stresses considering non-linear plastic deformation.

The yield function $f_\text {yield}$ is parameterized by two material-dependent properties: internal friction and cohesion. In the case of plastic deformation, we calculate an updated stress considering plastic yielding as

11 $$\begin{aligned} \sigma _{ij}^{n+1} = \kappa s_{ij}^\text {trial} + \sigma _{m}^\text {trial}\delta _{ij} \end{aligned}$$

where $\kappa$ is the yield factor, $\sigma _{m}^\text {trial}$ is the mean normal stress in the trial state and $s_{ij}^\text {trial}$ are the deviatoric stresses in the trial state. Finally, we need to convert the updated stresses $\sigma _{ij}^{n+1}$ back, from the nodal to the modal basis. We refer to Wollherr et al. (2018) for a detailed discussion on how parameters in (11) influence the yield function and on modeling and implementation of plastic yielding.

In summary, the plasticity kernels are executed after the predictor and corrector steps, and calculate the yield functions and plasticity deformation – both non-linear functions – in every element. We require kernels for transferring degrees of freedom between nodal and modal bases, expressed as tensor instructions, and a nonlinear kernel that modifies the stresses based on the (also nonlinear) yield function. For the latter kernels, we cannot use direct tensor contractions, but have to treat them separately when fusing the simulations (cf. Section 5.2).

### Optimized small matrix multiplication in SeisSol

The ADER-DG scheme, as summarized in Section 4.1, can be implemented entirely via many small matrix multiplications (or tensor contractions). The matrix sizes depend on the number of quantities in the vector $\textbf{q}$ and on the number of polynomial basis functions in each DG element (e.g., 56 for convergence order 6). Table 1 gives an overview of selected matrices for convergence orders 3 and 6, respectively.

**Table 1:** Matrix storage size of commonly used matrices in the predictor kernel, for convergence orders 3 and 6. In column *logical*, *size* denotes the true matrix dimensions; *storage* indicates whether the matrix is dense or sparse (with number of non-zeros). The columns *non-fused* and *fused* show the storage layout used in the respective implementation. Matrix sizes can increase due to alignment (“padding”) to vector lengths of SIMD registers (here for vector length 8); *block* indicates that a sparse matrix is stored as a dense block that contains all non-zeros

Matrix	order	logical		non-fused		fused	
		size	storage	size	storage	size	storage
$\textbf{Q}, \textbf{I}$	3	$10 \times 9$	dense	$16 \times 9$	dense (144)	$10\times 9$	dense (90)
$(\hat{\textbf{K}}^\xi )^T$	3	$10 \times 10$	sparse (7)	$16 \times 4$	block (64)	$16 \times 4$	sparse (64)
$\textbf{A}^*$	3	$9 \times 9$	sparse (24)	$9 \times 9$	sparse (24)	$9\times 9$	sparse (24)
$\textbf{Q}, \textbf{I}$	6	$56 \times 9$	dense	$56 \times 9$	dense (504)	$56\times 9$	dense (504)
$(\hat{\textbf{K}}^\xi )^T$	6	$56 \times 56$	sparse (294)	$56 \times 35$	block (1960)	$56 \times 35$	sparse (1960)
$\textbf{A}^*$	6	$9 \times 9$	sparse (24)	$9 \times 9$	sparse (24)	$9\times 9$	sparse (24)

The matrix or tensor operations are expressed as tensor contractions in Einstein summation notation, such as in eqs. (5), (7) or (8). The YATeTo package (Uphoff and Bader 2020) analyzes these tensor expressions, and generates optimized sequences of binary general matrix-matrix multiplications (GEMM), elementwise products and reductions out of them. The small-GEMM kernels are delegated to hardware-optimized libraries: LIBXSMM (Heinecke et al. 2016) and PSpaMM. The latter is developed as part of the SeisSol package^3^. Both libraries optimize performance by generating either just-in-time assembly (LIBXSMM) or inline assembly kernels (LIBXSMM and PSpaMM) that are better suited for small matrices than standard BLAS libraries. The latter are typically designed for larger matrices, and therefore underperform for the matrix sizes in SeisSol, due to overheads in memory accesses, loop management, and unoptimized handling of matrices of particular (fixed) size (Heinecke et al. 2016; Dongarra et al. 2017; Masliah et al. 2016; Yang et al. 2021; Yao et al. 2021; Abdelfattah et al. 2016).

To showcase the workflow of YATeTo, LIBXSMM and PSpaMM, we consider an operation of the form $R_{ij} = K_{ik}Q_{kl}A_{l j}$ (using Einstein summation notation), which reflects a core pattern in the calculations in (5), (7), and (8). $\textbf{K}$ is a comparably large and sparse matrix (cmp. Table 1), which is constant over all elements. $\textbf{A}$ is sparse, but small. $\textbf{Q}$ and the result $\textbf{R}$ are dense. YATeTo decomposes this chain matrix product into a sequence of binary products: a sparse-dense matrix multiplication $X_{il} = K_{ik}Q_{kl}$ followed by a dense-sparse matrix multiplication $R_{ij} = X_{il}A_{l j}$. Furthermore, YATeTo marks $\textbf{X}$ and $\textbf{R}$ as dense, since $\textbf{Q}$ is dense, and pads $\textbf{Q}$, $\textbf{X}$ and $\textbf{R}$ to SIMD length (see Table 1), to allow for more efficient code generation by LIBXSMM and PSpaMM.

In earlier work (Heinecke et al. 2014; Uphoff et al. 2004, e.g.), sparse-dense/dense-sparse multiplications were executed as dense-dense operations, if the sparsity (i.e., relative number of non-zero elements) of the involved matrices exceeded a certain threshold. We stress that for this work, we execute all sparse-dense/dense-sparse multiplications using sparse matrices and PSpaMM as code generator. Dense-dense operations are still executed via LIBXSMM.

Both LIBXSMM and PSpaMM generate hardware-optimized code (i.e., assembly instructions) that exploits SIMD instructions available on specific CPU architectures. The general implementation pattern for a dense-dense product (both matrices stored in row-major format) is to load consecutive elements of the left matrix into a SIMD register. The right matrix, in contrast, is loaded per-element, each element being broadcasted to all elements of a SIMD register to allow a subsequent SIMD operation (“fused multiply-add”; results accumulated in SIMD registers for the result matrix).

The difference in SIMD register handling for the two matrices requires different approaches to handling matrix sparsity efficiently: While dense-sparse products can be implemented by just removing all instructions and broadcasts of zero elements, sparse-dense products are much harder to implement efficiently.

For instructions such as $X_{il} = K_{ik}Q_{kl}$, we know that the stiffness matrix $K_{ik}$ is identical for all elements, is thus stored only once, and is expected to remain in low-level caches during computation. PSpaMM therefore employs a block-sparse (or padded sparse) approach with complete unrolling of element operations: $K_{ik}$ is decomposed over SIMD blocks, which means that starting from a row-major dense layout, we split into blocks of SIMD length, and only keep blocks that have at least one non-zero element. The compute kernels now omit instructions (and storage) for all blocks that are entirely zero, and for the remaining blocks mask out all known zero operations.

To summarize, we stress that the sparsity patterns of all matrices are known in advance and are exposed to the code generators YATeTo and PSpaMM. Efficient handling of sparse matrices adopts padding and dense blocks of SIMD length, which lead to additional operations being executed, but which are vastly superior to conventional sparse-matrix implementations in terms of time-to-solution (Heinecke et al. 2014, e.g.). We evaluate the respective padding overhead via measuring the gap between “hardware” (executed SIMD instructions) and “non-zero” (absolutely necessary) operations. Fused ensemble simulations will reduce this gap, particularly (see Section 5.1).

## Fused simulations

We now discuss the implementation of fused simulations to accelerate large-scale earthquake modeling. The key idea to accelerate the wave propagation kernels follows previous work by Uphoff (2020) and Uphoff and Bader (2020). We represent the degrees of freedom $Q_{ij}$ of multiple simulations $s=1,\dots ,S$ as a single rank-3 tensor $Q_{sij}$ stored in each element. To update $Q_{sij}$, the ADER-DG scheme proceeds as described in Section 4.1, essentially adding an additional dimension *s* to all numerical steps. The adjusted Cauchy–Kovalevskaya procedure (5) then reads

12 $$\begin{aligned} {\begin{matrix} D^{p + 1}_{sij} :=& \hat{K}_{im}^\xi D^p_{sml}A_{lj}^* + \hat{K}_{im}^\eta D^p_{sml}B_{lj}^* + \hat{K}_{im}^\zeta D^p_{sml}C_{lj}^* \qquad D^0_{sij} := Q_{sij}, \\ \text {and~} {I}_{sij} =& \sum _{p = 0}^{P} \frac{\Delta t^{p+1}}{\left( \delta + 1\right) !} {D}^p_{sij}. \end{matrix}} \end{aligned}$$

The local corrector part (7) and the final corrector step (8) become

13 $$\begin{aligned} {\begin{matrix} Q_{sij}^{n+1, *} & = Q_{sij}^{n} + \tilde{K}_{im}^\xi I_{sml}A_{lj}^* + \tilde{K}_{im}^\eta I_{sml}B_{lj}^* + \tilde{K}_{im}^\zeta I_{sml}C_{lj} ^* \\ & - \frac{1}{\left| J\right| } M^{-1}_{il}\biggl (\sum _{f=1}^4\left| S_f\right| F^{-,f}_{lm}I_{smn}\hat{A}^+_{nj}\biggr ) \end{matrix}} \end{aligned}$$

14 $$\begin{aligned} \text {and}\quad Q_{sij}^{n+1} = Q_{sij}^{n+1, *} - \frac{1}{\left| J\right| } M^{-1}_{il} \biggl (\sum _{f=1}^4 \left| S_f\right| {F}^{+,i}_{lm} I^{f, -}_{smn}\hat{A}^-_{nj}\biggr ). \end{aligned}$$

For implementation, the key question is whether to store the tensor $Q_{sij}$ with the “fastest-running” index *s* , i.e. as $(Q_{1,1,1}, \dots , Q_{S,1,1}, \dots , Q_{1,N,M}, \dots , Q_{S,N,M})$ in each cell, or to store the fused data sets sequentially – resulting in a cell-local data layout $(Q_{1,1,1}, \dots , Q_{1,N,M}, \dots , Q_{S,1,1}, \dots , Q_{S,N,M})$. Here, *N* denotes the number of basis functions and *M* the number of quantities. As described in Section 5.1, having the “fastest-running” index *s* is favorable for the fused wave propagation kernels, moving from batched matrix to batched tensor kernels. In contrast, as outlined in Section 5.2, the dynamic rupture and plasticity computations are nonlinear and cannot be expressed via batched matrix or tensor kernels alone, but require separate treatment.

### Fusing the wave propagation kernels

The fused wave propagation kernels are again expressed in the YATeTo domain-specific language (cf. Section 4.4), following Einstein convention as in eqs. (12) to (14). Hence, kernels essentially need to add one index for the multiple simulations. Code generation now follows the existing YATeTo implementation (without changes necessary compared to single simulations) and uses the same PSpaMM implementation as the backend. However, as described in this subsection, different small sparse-dense and dense-sparse matrix multiplications are generated that better exploit the SIMD registers of CPU hardware and require less padding for storage.

As in Section 4.4, we consider an example expression from (13), which we write as $R_{sij} = K_{ik} Q_{skl} A_{l j}$. Again, YATeTo will decompose this tensor operation into two steps, $X_{sil} = K_{ik} Q_{skl}$ and $R_{sij} = X_{sil}A_{l j}$. Here, $Q_{skl}$ as well as $R_{sij}$ and $X_{sil}$ are dense tensors of size $S \times N \times M$, whereas $K_{ik}$ and $A_{l j}$ are sparse matrices. Next, YATeTo transforms the first operation into a loop (with loop variable *l*) over GEMM operations: fixing *l* and contracting over *k*, each $X_{sil} = Q_{skl} K^T_{ki}$ becomes a dense-sparse matrix multiplication, executed by PSpaMM. If *S* is selected as a multiple of the SIMD length, this does not cause any sparse padding penalty (*s* being the fastest-running index) – in contrast to the non-fused situation. The second operation, $R_{sij} = X_{sil}A_{l j}$, is computed by a single dense-sparse GEMM that contracts over *l* and collapses the *s* and *i* dimensions. PSpaMM executes this dense-sparse multiplication as described in Section 4.4, which does not cause any padding due to sparsity of $A_{l j}$, and also avoids padding in $X_{sil}$ and $R_{sij}$, as the collapsed row index *si* is a multiple of the SIMD length (*S*).

This “loop over GEMM”-implementation for $X_{sil} = Q_{skl} K^T_{ki}$ and collapsed dense-sparse-GEMM implementation for $R_{sij} = X_{sil}A_{l j}$ has the following impacts on kernel performance (always assuming that *S* is chosen a multiple of the SIMD length):

- We avoid “dense padding” to SIMD length for the tensor of unknowns $Q_{skl}$: for example, assuming a SIMD length of 8 and order 3, $\textbf{Q}$ is padded for serial executions from size $10\times 9$ to size $16\times 9$ (compare Table 1). Fused simulations use an $S \times 10\times 9$ tensor without any padding. We thus also reduce the costs for fetching the $Q_{kl}$/$Q_{skl}$ from main memory – by a factor of $16/10 = 1.6$ in the given example.
- We avoid “sparse padding” for the operation $X_{sil} = Q_{skl} K^T_{ki}$: We thus increase the fraction of non-zero operations compared to hardware operations.
- Especially at low order, sparse and dense padding together increase the arithmetic intensity of the kernels.
- Collapsed loops in the operation $R_{sij} = X_{sil}A_{l j}$ and differences in register usage for unrolled matrix vs. tensor operations may lead to further performance improvements.
- On the other hand, fused simulations might compromise one of the central assumptions in our ADER-DG optimization, which is that element-local operations fit into low-level caches. Especially for high polynomial order, this assumption will be violated if *S* is chosen too large. For example, at order 6 and with $S=16$, the tensor $Q_{skl}$ is already of size $16 \times 56\times 9$, equivalent to 8,064 elements and approximately 64 KB for double precision, which already exceeds the size of the level-1 caches of our architectures (compare Section 7.1).

### Fusing the non-linear kernels

As indicated in Sections 4.2 and 4.3, the dynamic rupture and plasticity kernels are non-linear, and we cannot directly utilise the tensor structure to “fuse” the simulations. We therefore employ and compare two strategies, called *looped* and *interleaved*, which differ in their respective implementation effort.

***Looped strategy:***

In the looped strategy, we strive to keep the implementation of the kernels unchanged. We change the data layout from $(Q_{1,1,1}, \dots , Q_{S,1,1}, \dots , Q_{1,N,M}, \dots , Q_{S,N,M})$ to $(Q_{1,1,1}, \dots , Q_{1,N,M}, \dots , Q_{S,1,1}, \dots , Q_{S,N,M})$, i.e., from $Q_{sij}$ to $Q_{ijs}$ format, to enable a straightforward outer loop over the simulation index *s* (i.e., over the *S* problems). For all problems *s*, each $Q_{ij}$ tensor is thus processed independently for dynamic rupture and plasticity, using the existing kernels. Afterwards, the data structures are transformed back to the original $Q_{sij}$ format. The algorithm is summarized as:

- Initialize a temporary data structure to store the transposed data structure.
- Copy the data structure into the temporary data structure $\hat{\textbf{Q}}$ with ordering $\hat{Q}_{ijs}$.
- Perform for each simulation $s = 1, \dots , S$:
  - Derive the memory handle of simulation *s* by offsetting the base pointer of $\hat{\textbf{Q}}$ according to the simulation index.
  - Perform the (existing) non-linear kernel on this data.
- Copy the computed data from the temporary data structure into the original data structure with format $Q_{sij}$.

In the case of dynamic rupture, this algorithm is executed for all faces with a dynamic rupture interface. For plasticity, it is executed for all elements. The algorithm incurs an overhead of transposing the degrees-of-freedom tensor twice, which can degrade performance, especially in higher-order simulations.

***Interleaved strategy:***

In the interleaved strategy, we avoid the computational cost of changing the data layout during the two non-linear kernels, but invest additional implementation effort. We keep the tensor layout $Q_{sij} = (Q_{1,1,1}, \dots , Q_{S,1,1}, \dots , Q_{1,N,M}, \dots , Q_{S,N,M})$ as is, with *s* as the fastest-running index. Within the dynamic rupture and plasticity implementation, all linear operations outlined in Sections 4.2 and 4.3 – i.e., evaluating the 3D solution at time quadrature points from the element-local space-time predictor solution, projection of 3D space solution to 2D dynamic rupture faces at each time quadrature point, and transferring degrees of freedom between nodal and modal bases in plasticity – are expressed as tensor instructions (adding the index *s*) and handled by the YATeTo code generation. To perform the non-linear operations, we replace the loop over quadrature points *i* by a loop over two tensor indices *s* and *i*, i.e., simulation and quadrature points. For plasticity, this implies calculation of the yield function and correcting the stresses according to the yield function. For dynamic rupture, it includes checking whether the fault is locked, iterating for the friction law, and calculating the slip rate. Note that in order to vectorize over the *s*-*i*-loop, we need to enforce a uniform number of iterations for any iterative part of these calculations. For complicated friction laws, such as rate-and-state, this induces an overhead, as we have to wait for the slip-rate iterations of all simulations to converge. Similarly, a cell will compute the plasticity yield for all simulations, even if only a single simulation yields. Hence, we introduce some algorithmic overhead despite avoiding memory transfers to change data layout.

### Validation of correctness

We validate the correctness of the fused-simulations implementation against the SCEC TPV13 community benchmark (Harris et al. 2018). TPV13 is a 3D benchmark for dynamic rupture with off-fault plastic yielding^4^. It prescribes a 60° dipping planar, normal fault ($30\times 15$ km) embedded in a homogeneous halfspace. The fault reaches the Earth’s surface and extends up to $\approx$13 km in depth. The off-fault rheology is non-associative Drucker-Prager visco-plastic (cmp. Section 4.3). Depth-dependent initial stress conditions are prescribed on and off the fault. The linear slip-weakening friction law is used. Strongly super-shear rupture conditions are assumed. Rupture is initiated in a square nucleation area (side length 3 km), which is centered at $(0, -12)$ km (in fault coordinates) at a depth of $\approx$10.4 km. We compute on a mesh with $\approx$1.4 million elements, with $\approx$25.5 thousand dynamic rupture faces (i.e., computing dynamic rupture). For the fused simulations, we vary the coefficient $\mu _s$ for static friction inside the nucleation patch. For one simulation, we set it to obtain no rupture at all.

**Fig. 1:**
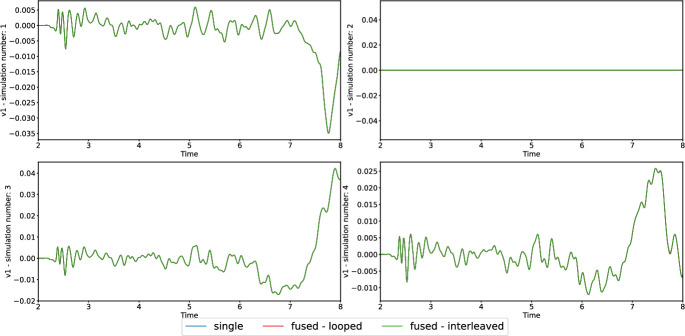
Validation of TPV13 results using single precision. Each plot compares the results of fused simulations with different strategies with their serial counterparts, revealing no visible discrepancy. The parameters of simulation 2 (top right) were set such that there is no rupture, to validate this case, as well. The time axis is clipped before 2 s of simulation time, as no waves are observed there, yet

**Fig. 2:**
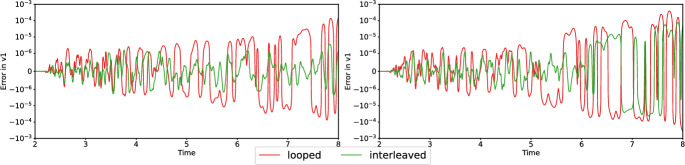
Absolute errors between different strategies of fused and serial simulations for simulations 3 and 4 of Figure 1, using single precision. The y-axis is plotted using a logarithmic scale for magnitudes greater than $10^{-6}$

To verify that fused simulations do not produce major differences in the results, we compared particle velocities at different receiver locations for fused and single simulations, using the looped and interleaved strategies introduced in Section 5.2. Figure 1 plots the velocity $v_1$ at receiver $\left( 0.0, 826.795, -300.0\right)$ m, Figure 2 shows the corresponding absolute errors for simulation numbers 3 and 4, all computed in single precision. We observe errors in the order of $10^{-4}$ for both strategies.

**Fig. 3:**
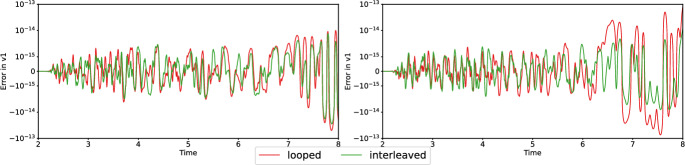
Absolute errors between different strategies fused and serial simulations for simulations 3 and 4 of Figure 1, using double precision. The y-axis is plotted using a logarithmic scale for magnitudes greater than $10^{-15}$

We repeat the validation using double precision, which reduces the maximum absolute error to $10^{-13}$ for both the strategies – see the plots in Figure 3.

We observe close agreement of the solutions, with negligible floating-point errors in the case of double precision. The errors are higher (in the range of $10^{-4}$) with single precision, which we attribute to a high sensitivity of dynamic rupture simulations w.r.t. the selected precision. Differences in rounding can be due to different execution orders of operations and to different intermediate rounding of floating-point values in sequences of SIMD operations. However, our results indicate that the fusion of simulations does not have a significant impact on accuracy.

## Launching fused ensemble simulations in UQ workflows with UM-Bridge

As outlined in Section 2, we rely on UM-Bridge to implement complex UQ workflows with SeisSol. UM-Bridge is a software interface between UQ algorithms (a *client*) and numerical simulators (a *model*), and enables any client to request simulation runs for a UQ workflow (Seelinger et al. 2025). UM-Bridge schedules simulation runs on a compute cluster in a way that is entirely transparent to the client. SeisSol provides an UM-Bridge wrapper as a model. This section describes how we extend this setup to support fused simulations. The key challenge is that the UQ side remains oblivious to fusing simulations, making fused simulations a drop-in replacement for simulations within *any* UM-Bridge enabled workflow that allows sufficient parallelism.

### Batching UM-Bridge model queries

In order to transparently create batches of parameters for fused simulations, we introduce a microservice-style component in between UQ and simulator. To the UQ side, this batcher^5^ appears as an UM-Bridge model taking a single parameter vector and returning a single model output, and is therefore entirely equivalent to non-fused simulation runs. Internally, it collects batches of parameters, passes entire batches to the UM-Bridge HPC load balancer (which spins up SLURM jobs with SeisSol as needed), unpacks batched model outputs, and returns them individually to the respective client request. The simulator is only concerned with processing one parameter batch at a time, and is therefore oblivious to how parameter batches are assembled or how the UQ side is parallelized. Collection and execution of batches are fully thread-parallel, ensuring the batcher remains permanently responsive.

Note that if, within a specified time period, not enough incoming requests arrive to fill the prescribed batch size, we pad the existing request parameters with nominal parameters to meet the full batch size. This guarantees timely execution of simulations but, of course, wastes some of the fused simulations in a batch.

The resulting design enables dynamic and asynchronous batched parameter evaluations into fused ensembles with improved computational efficiency, while maintaining compatibility with the UM-Bridge API and minimal intrusion on the simulator code. Additionally, we include retry handling in the batcher to mitigate potential instabilities in large-scale HPC environments.

### SeisSol UM-Bridge server

To enable seamless integration with UM-Bridge and support for fused simulations, we extended SeisSol’s UM-Bridge server. When a client request (from MLDA in our case) arrives, the wrapper generates input files for SeisSol through the Inja templating engine (https://pantor.github.io/inja/), executes the simulation, and returns misfit values computed by a Python postprocessing script.

Depending on the simulation configuration (e.g., order 3 or 4), the wrapper dynamically selects the appropriate binary for either fused or serial simulations. Environment variables and system-level settings are adjusted depending on the computing platform, for example with specific configurations tailored for the CPU architecture. The generated input files (incl. parameters, fault details, etc.) are placed into a scratch directory, identified by the SLURM job ID to separate files of concurrent runs. The misfit computation to calculate the loglikelihood for the MLDA algorithm is performed externally via a Python script, and results are captured and parsed from standard output. The resulting workflow proceeds as follows:

1. The client sends simulation parameters to the batcher.
2. The batcher collects the simulation parameters, batches them as per the configuration and sends the batch to the UM-Brige server.
3. The UM-Bridge HPC loadbalancer receives the request and spans SLURM jobs.
4. The UM-Bridge wrapper for SeisSol inside the SLURM jobs processes the request:
  Produces input files (via template generation) based on the received parameters.
  Executes the appropriate SeisSol binary on the generated files.
  Evaluates the misfit (via a Python script) between the written simulation output and the observed data.
5. The resulting misfit values are sent back to the batcher.
6. The batcher disaggregates the results and returns them to the client.

This setup enables efficient, scalable black-box evaluations of SeisSol via UM-Bridge clients and allows us to separate fused simulations from serial simulations for different configurations.

## Performance of fused earthquake simulations

### Architectures

For our experiments and performance studies, we used three different CPU-based supercomputers with different architectures, with the following specifications:

- Vista^6^ (Texas Advanced Computing Center, TACC), in its Grace-Grace subsystem, offers 256 nodes equipped with an NVIDIA Grace-Grace CPU Superchip that combines two Arm CPUs, each with 72 cores, clocked at 3.4 GHz and including 64 KB of L1 cache. Each node has 237 GB of LPDDR DRAM memory with a main memory bandwidth of 850 GiB/s and a peak performance of 7.1 TFlop/s (using double precision).
- Frontera^7^ (TACC) combines 8,368 compute nodes with two Intel Xeon Platinum 8280 (Cascade Lake) CPUs, each with 28 cores, clocked at 2.7 GHz and including 32 KB of L1 cache. Each node has 192 GB of DDR-4 memory with a main memory bandwidth of 282 GB/s and a peak performance of 4.8 TFlop/s (double precision).
- SuperMUC-NG^8^ (Leibniz Supercomputing Centre, LRZ) offers 6,336 compute nodes (“thin nodes” partition) equipped with two Intel Xeon Platinum 8174 (Skylake) CPUs, each with 24 cores, clocked at 2.7 GHz and including 32 KB of L1 cache. Each node has 96 GB of memory with a main memory bandwidth of 102.4 GB/s and a peak performance of 4.15 TFlop/s (double precision).

While the SIMD width of Vista’s Arm CPUs is 16 bytes (2 double-precision values per SIMD register), it is 64 bytes (8 doubles) for the Intel CPUs on Frontera and SuperMUC-NG. We can thus efficiently fuse 2 double-precision simulations, or any multiples of 2, on Vista, but are restricted to multiples of 8 on Frontera and SuperMUC-NG, i.e., fuse 8, 16, ... simulations without wasting performance.

### Performance evaluation of fused wave-propagation kernels

**Fig. 4:**
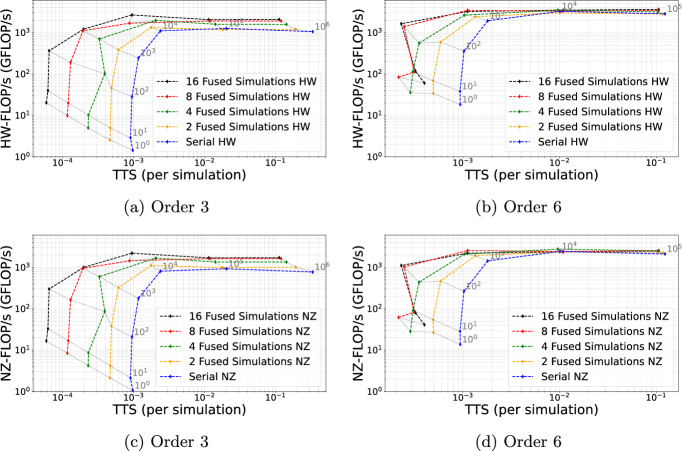
HW-FLOP/s (plots a and b) and NZ-FLOP/s (c and d) plotted against the time-to-solution (TTS) per simulation, when running the SeisSol proxy on Vista (Grace-Grace) with and without fused simulations. We increase the problem size from 1 to $10^6$ elements (in steps of factor 10; grey lines connect identical problem sizes). For small element counts, the kernel launch time dominates (plots growing vertically), whereas for larger element counts, the throughput dominates (plots plateauing)

As a first step, we examine the influence of fused simulations on the performance-dominating kernels for seismic wave propagation. SeisSol-Proxy is a mini app that executes the key SeisSol compute kernels on arbitrary data to allow detailed performance studies. In Figure 4, we compare the floating-point operations achieved per second (FLOP/s) achieved for single vs. fused simulations on a single Grace-Grace node of Vista. While we consider all FLOP/s issued in the hardware units (“Hardware FLOP/s”: HW-FLOP/s) in subfigures (a) and (b), we display the actual FLOP/s contributing towards the solution (“Non-Zero FLOP/s”: NZ-FLOP/s) in subfigures (c) and (d). The NZ-FLOP/s exclude any operations from the HW-FLOP/s done due to SIMD register padding or handling a sparse as dense matrix. Hence, HW-FLOP/s consider the actual number of computations performed by the computer, while NZ-FLOP/s are the number of useful computations done.

We executed only the wave propagation kernels (local element updates and neighbor kernels) using 1 MPI rank per node and 143 OpenMP threads per rank. We pinned the OpenMP threads to cores in a 1:1 fashion, using the  option with a  binding, thus leaving one core to be used for a communication thread.

We plot the achieved HW-FLOP/s and NZ-FLOP/s vs. the time to solution required to update the elements, where we increase the number of elements by a factor of 10 from each test run to the next.

We observe for all simulations (fused and non-fused) that the time-to-solution first stagnates, as the launch of kernels dominates the computing time. Note also that OpenMP parallelism (here with 143 threads) can only be exploited for more than 100 elements. All simulations then plateau, eventually reaching the full kernel performance for a large ($>10^4$) number of elements. The fused-simulation kernels plateau earlier, though, and at a higher performance than serial simulation. For discretization order 3, we observe an increase of HW-FLOP/s by a factor of up to 1.97 and NZ-FLOP/s by a factor of up to 2.21 for 16 fused simulations. The improvement is much smaller for order 6: factor 1.28 for HW-FLOP/s and 1.7 for NZ-FLOP/s of 1.17. With order 6, we achieve a maximum performance of 3.5 TFLOP/s using 16 fused simulations, which is close to 50% of the peak performance; for order 3, we reach 2.0 TFLOP/s, representing roughly 28% of the peak performance.

These results indicate that in addition to the five aspects already mentioned in Section 5.1, there are two further issues that impact performance: Due to the short SIMD length of the Grace-Grace superchip, dense padding and sparse padding should lead to a much smaller effect than the observed $\approx$2$\times$ speedup for the memory-bound situation of order 3. We attribute the larger speedup to memory latency effects, i.e., that larger blocks of memory (consisting of several fused element-local data structures) are transferred more efficiently on Grace-Grace, leading to an improved memory access pattern. Second, we observe from the gray lines in Figure 4 that for a fixed cluster size, the improvement is much larger for small clusters: for order 3, the NZ-FLOP/s increased by a factor of 14.43 for $10^2$ and 5.57 for $10^3$ elements (comparing serial execution with 16 fused simulations). Here, the launch overhead for OpenMP kernels comes into play, and we have to consider that with 143 threads, each thread will only have a few elements assigned. Launch times are thus large compared to processing times, and launch overheads are amortized when using many fused simulations.

The performance benefit for small clusters is particularly relevant when running SeisSol with local time stepping (LTS). On realistic meshes with aggressive mesh refinement and complicated geometries, we often encounter grid cells with bad element shapes that enforce a small local time step. SeisSol’s LTS algorithm combines elements with similar time step limits into LTS clusters, which are updated with a uniform time step (Breuer et al. 2016; Uphoff et al. 2004). Figure 4 illustrates that small LTS clusters suffer from a substantial performance reduction.

### Performance for medium-size problems – TPV-13 and Searles Valley Case Studies

Next, we test the performance and scalability of fused simulations on Frontera and Vista, focusing first on problems of moderate size, to reflect the behavior of model evaluations that form the bulk of executions in an MLDA workflow. We run eight different simulations of the TPV13 benchmark (cmp. Section 5.3) with varying coefficient $\mu _s$ of static friction, first as serial and then as fused simulations, and compare the execution times for an increasing number of nodes. We use two MPI ranks per node (i.e., one rank per socket) with 27 OpenMP threads per rank for Frontera and 71 threads per rank for Vista, hence, again reserving one thread per rank for the communication. For pinning threads to cores, we use the approach described in Section 7.2. As a performance metric, we use “simulations per node hour”, which is the relevant metric for large workflows: it specifies how many simulation runs we can execute for a given amount of compute resources. We performed all scaling tests for 1.0 s of simulated time and extrapolated the number of simulations per node hour to 8.0 s. In all our performance and scaling tests in Sections 7.3 and 7.4, we ignore the mesh reading, model initialization, and I/O.

**Fig. 5:**
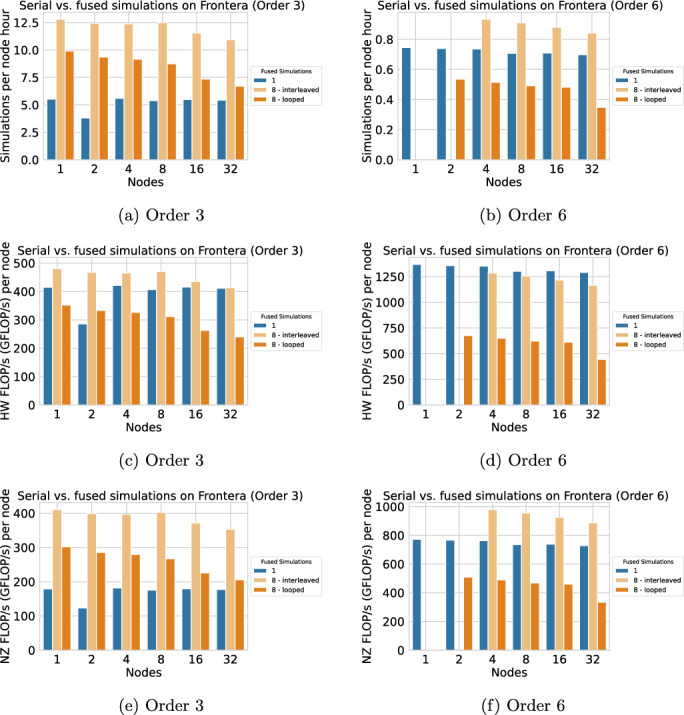
Performance for the TPV-13 benchmark on Frontera, comparing single (blue) with eight fused (orange) simulations with fused vs. interleaved strategy at discretization orders 3 and 6. Subfigures (a) and (b) illustrate simulation throughput in terms of simulations per node-hour, (c) and (d) raw hardware performance (hardware FLOP/s), (e) and (f) computational efficiency (non-zero FLOP/s)

In Figure 5, we plot the simulations per node hour achieved on Frontera for discretization orders 3 and 6. Since the configuration of 1 node with order 6, and 2 nodes with order 6 with “interleaved” strategy did not fit in memory, the data points are missing in these scenarios. For order 3 and the interleaved strategy, we observe a speedup of 2.3, resulting in a 57% reduction in compute time, on a single node. As the node count increases, the speedup decreases slightly, reaching 2.0 (i.e., 50% reduction in node hours) on 32 nodes. For this memory-bound situation, the speedup is in good agreement with what we can expect from reduced memory transfers, due to avoiding dense padding (compare HW-FLOP/s and NZ-FLOP/s). Speedups for the looped strategy are substantially smaller, from 1.8 on a single node to 1.2 on 32 nodes. For order 6, we observe a performance drop for the “looped” strategy, particularly visible in the low HW-FLOP/s, which we attribute to element-local data structures no longer fitting in the L1 cache, and to the overhead of transposing them. For the “interleaved” strategy, we only see a small reduction in HW-FLOP/s and thus still speedups w.r.t. NZ-FLOP/s and simulations per node hour: from 1.21 on 4 nodes to 1.17 on 32 nodes. For both orders, we observe lower parallel efficiency in the fused simulations, the effect being particularly strong with the looped strategy and with lower order. One possible cause of this could be the load imbalance in dynamic rupture kernels. Their computational effort can only be roughly estimated for the partitioning, such that wrong estimates affect the load imbalance more strongly for fused simulations.

**Fig. 6:**
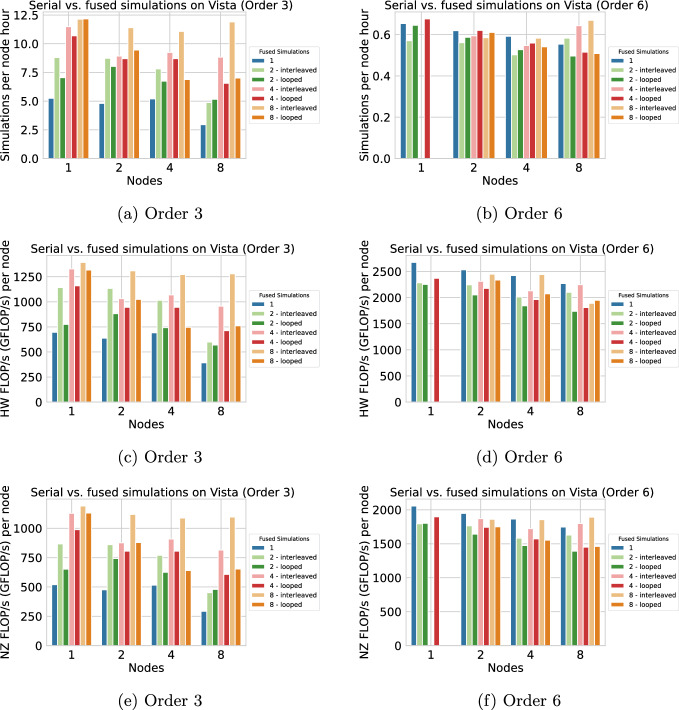
Performance for the TPV-13 benchmark on Vista, comparing single (blue) with 2, 4, or 8 fused simulations (all for discretization orders 3 and 6). Subfigures (a) and (b) illustrate simulation throughput in terms of simulations per node-hour, (c) and (d) raw hardware performance (hardware FLOP/s), (e) and (f) computational efficiency (non-zero FLOP/s)

In Figure 6, we plot the simulations per node hour achieved on Vista, again for orders 3 and 6. For order 6 on a single node, memory was not sufficient for the “interleaved” configuration of 4 or 8 fused simulations, and the “looped” configuration of 8 fused simulations. The respective data points are missing.

For order 3, using 8 fused simulations and the interleaved strategy, we achieve speedups of 2.3 on 1 node and 4.0 on 8 nodes (for simulations per node hour). Fused simulations are, in contrast, slower for order 6, though by a small margin. While results on multiple compute nodes are rather volatile, we notice a general trend of obtaining better parallel efficiency for fused simulations. We attribute this to larger problem sizes per node and a stronger impact of size-dependent performance due to local time stepping (LTS), as observed in Figure 4. Comparing the results on Frontera and Vista, we notice a much stronger discrepancy between HW-FLOP/s and NZ-FLOP/s on Frontera, which is mainly an effect of the shorter SIMD length on the Arm CPUs, such that serial simulations suffer less from padding overhead. Also, the differences between looped and interleaved strategy are smaller on Vista, indicating a smaller impact of limited cache size (see the results for the looped strategy; L1 caches are larger on the Grace-Grace superchip).

**Fig. 7:**
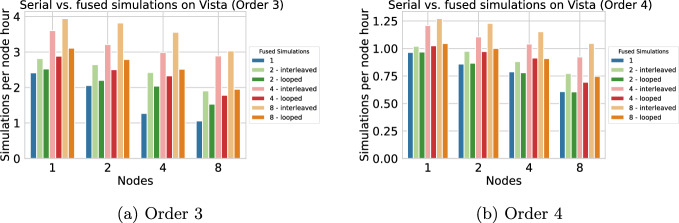
Extrapolated simulations per node hour achieved for the Searles Valley earthquake simulation on Vista (Grace-Grace), comparing single execution (blue bars) with 2, 4, or 8 fused simulations (all for discretization orders 3 and 4). The scaling tests have been performed for 6.0 s of simulated time. The number of simulations per node hour is extrapolated to consider a simulation time of 20.0 s

We now consider dynamic rupture simulations of the 2019 $\hbox {M}_w$ 6.4 Searles Valley earthquake (Figure 9a), which involves rupturing a complex fault system comprising conjugate non-planar segments. This scenario is the relevant scenario for our Bayesian inference in Section 8. The simulations integrate nonlinear frictional interactions on the fault (Dunham et al. 2011) and the propagation of waves generated from the breaking fault. The computational domain was discretized with a mesh of 4.02 million elements, ensuring adequate resolution of both the fault-zone processes and seismic wave propagation (Kruse et al. 2024). In Figure 7, we plot the simulations per node hour achieved on Vista, for orders 3 and 4, which are relevant for our Bayesian inference in Section 8. We observe maximum speedups of 2.8 for order 3 and 1.7 for order 4, both on 8 nodes with 8 fused simulations using the “interleaved” strategy. Due to a higher number of dynamic rupture faces, we see a stronger performance overhead for the looped strategy in this scenario. We also observe better scaling with fused simulations, probably because the effects of size-dependent performance due to small time clusters (as observed in Figure 4) are more prominent with higher node counts.

### Performance for large problems – Kahramanmaraş scenario

We now test the performance and scaling of fused simulations for a large-scale production scenario. We simulate the 2023 Kahramanmaraş, Turkey, $\hbox {M}_w$ 7.8–7.7 earthquake doublet, using models by Gabriel et al. (2023) and Jia et al. (2023), which involved complex rupture dynamics across numerous fault segments. This scenario models elastic wave propagation with Drucker-Prager plastic deformation and a linear slip weakening friction law (cmp. Sections 4.2 and 4.3).

**Fig. 8:**
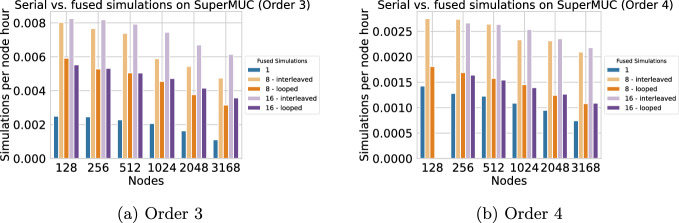
Extrapolated simulations per node hour achieved for the Turkey scenario on SuperMUC-NG, comparing single execution (blue bars) with fused simulations with 8 or 16 fused simulations (all for discretization orders 3 and 4). The scaling test was performed for a simulation time of 1 s. Simulations per node hour are extrapolated for a simulation time of 150 s

We use a mesh with approximately 175 million elements, which leads to 15.75 billion degrees of freedom at discretization order 3, and 31.5 billion degrees of freedom at order 4. For the performance study, we truncated the simulation to the first 1.0 s of the event and extrapolated the number of simulations per node hour accordingly. Tests were performed on SuperMUC-NG with one MPI rank per node with 94 OpenMP threads per rank ($2\times 47$ due to hyperthreading on SuperMUC-NG), again reserving one core per rank for the communication and pinning it to the last core as described in Section 7.2. We plot the extrapolated simulations per node hour for orders 3 and 4 in Figure 8. Since the configuration with 128 nodes, order 4, and 16 fused simulations did not fit in memory, this data is missing in Figure 8b.

On SuperMUC-NG, configurations with both 8 and 16 fused-simulations performed better than serial simulations for both orders 3 and 4. With 16 simulations and order 3, we observe speedups ranging from 3.3 on 128 nodes to 5.5 on 3184 nodes (half of SuperMUC-NG) using the interleaved strategy. For order 4, we observe speedups of 2.1 on 256 nodes to 2.9 on 3168 nodes (16 simulations, interleaved). For both orders, the looped strategy performs worse than the interleaved strategy owing to the higher number of memory transfers required. In terms of parallel efficiency, fused simulations increase the work load per MPI rank as well as the communication volume by a factor *S*. The improvement in scalability for fused simulations suggests that on SuperMUC-NG, we profit more from the increased workload.

We performed the same scaling test on the Frontera supercomputer during a *Texascale Day*, which enabled full-machine runs. At this time, only the looped strategy was available. Due to performance fluctuations across compute nodes, we only obtained reliable results for selected setups, which we therefore present in table form – see Tables 2 and 3. For order 3, we observed a peak non-zero performance of 870 TFLOP/s (on 3584 nodes) with 8 fused simulations and a maximum speedup of 1.88 relative to serial execution. For order 4, we observed the peak non-zero performance with serial simulations, at 1.05 PFLOP/s on 3584 nodes. Fused simulations with the “looped” strategy only reached a relative performance between 0.83 and 0.91.

Overall, the performance results for the Kahramanmaraş scenario agree well with those for the smaller TPV13 benchmark. The gains from fused simulation are higher, especially at order 3, which we attribute to a more complex LTS-cluster situation, with more time-step-size levels and smaller LTS clusters.

**Table 2:** Performance comparison of fused and serial simulations for the Turkey scenario on Frontera for order 3. “Relative performance” states the ratio of simulations per node hour between fused and serial execution

Nodes	Fused	Simulations per	HW FLOP/s	NZ FLOP/s	Relative
	simulations	$10^3$ node hours	[PFLOP/s]	[PFLOP/s]	Performance
	1	3.00	1.22	0.467	1
3584	8	5.66	1.02	0.870	1.88
	16	4.92	0.918	0.756	1.64
	1	3.34	1.17	0.448	1
3072	8	5.73	0.886	0.745	1.72
	16	5.77	0.924	0.760	1.73
	1	3.48	1.01	0.386	1
2560	8	5.10	0.657	0.559	1.47
	16	6.13	0.817	0.673	1.76

**Table 3:** Performance comparison of fused and serial simulations for the Turkey scenario on Frontera for order 4. “Relative performance” states the ratio of simulations per node hour between fused and serial execution

Nodes	Fused	Simulations per	HW FLOP/s	NZ FLOP/s	Relative
	simulations	$10^3$ node hours	[PFLOP/s]	[PFLOP/s]	Performance
	1	1.84	2.49	1.05	1
3584	8	1.58	1.12	0.904	0.86
	16	1.53	1.14	0.874	0.83
	1	1.86	1.80	0.757	1
2560	8	1.69	0.856	0.690	0.91
	16	1.60	0.850	0.653	0.86

## Fused simulations in UQ workflows

In this section, we demonstrate that fused ensemble simulations can yield performance gains in actual, complex UQ workflows. Specifically, we perform Bayesian parameter inference for a complex earthquake model using the Multilevel Delayed Acceptance (MLDA) method (Lykkegaard et al. 2023; Kruse et al. 2024) outlined in Section 2.

### Bayesian inversion through parallel MLDA

As a Bayesian approach, MLDA considers inverse problems in which the available data are noisy measurements of the output of a PDE model run with an unknown parameter $\theta$. It combines prior knowledge about the parameter with a likelihood derived from the assumed observation noise to obtain a posterior distribution $\pi$. This posterior distribution expresses which parameter values are more likely, given the observed data and prior information. In general, Markov Chain Monte Carlo (MCMC) methods generate a sequence of correlated parameter samples by proposing candidates and accepting or rejecting them based on how well they fit the data. Standard MCMC methods are often very expensive because each acceptance decision requires a PDE solve to evaluate the model-based likelihood, and many samples are usually needed to obtain reliable, effectively independent draws. Multilevel MCMC algorithms, such as the MLDA algorithm, decrease the high costs of such evaluations by employing a hierarchy of models with different accuracy-cost tradeoffs (Dodwell et al. 2019).

In this work, we restrict ourselves to an MLDA hierarchy of two levels with corresponding posterior densities $\pi _1$ and $\pi _2$. The coarse level (using a lower polynomial order in SeisSol) is cheaper to compute, but only yields coarse approximations of the posterior. The fine level (higher order in SeisSol) is more expensive, but more accurate – and the fine level also determines the final computed posterior: $\pi = \pi _2$. MLDA generates high-quality proposals at the fine level by spawning an MCMC chain on the coarse level at the current state, and using the final coarse-level sample as a proposal for the fine level. MLDA is thus inherently sequential. A method of parallelization, though, is to generate multiple independent MLDA chains and combine their results. This is valid since all chains sample the same posterior; however, since each chain requires a burn-in period and mixing, parallel chains have diminishing returns. In Kruse et al. (2024), we introduced within-chain parallelization through prefetching to mitigate that limitation. For this particular inversion study, we restrict ourselves to parallel chains only, i.e., without any prefetching, as SeisSol scales well enough not to reach the regime where prefetching pays off.

### Bayesian parameter inversion – Searles Valley earthquake

**Fig. 9:**
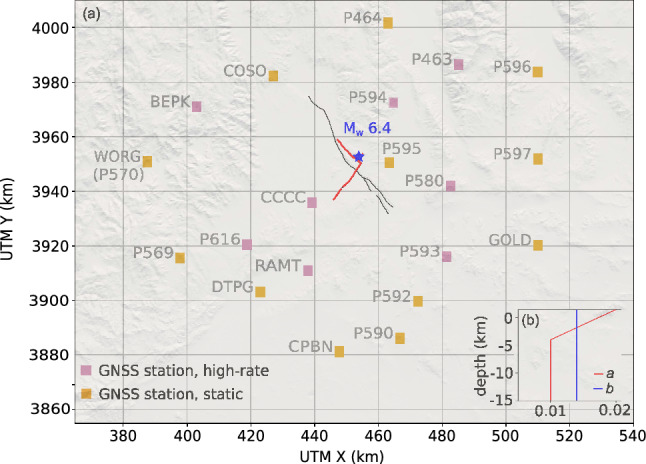
Data and model setup ($M_w$ 6.4 Searles Valley earthquake) for the inversion scenario. (a) Map view of the GNSS stations that constrain the inversion. Pink boxes denote stations where processed displacement time histories are available from Melgar et al. (2020); orange boxes are stations where we only use co-seismic static displacement from Floyd et al. (2020). All stations are located within a distance of 100 km to the epicenter (blue star). The red curve marks the fault trace ruptured by the earthquake. (b) Depth dependency of the friction parameters *a* and *b*

We consider the Searles Valley earthquake as mentioned in Section 7.3 for our Bayesian inversion. As a parameter of interest, we select one physically important parameter $a-b$ for friction that controls the spontaneity of the rupture dynamics (Dunham et al. 2011). In rate-and-state friction laws (Dieterich 1979; Ruina 1983), *a* and *b* jointly determine how the friction strength depends on the slip rate between two sides of the fault (direct effect vs. evolution effect). Earthquakes nucleate on faults with $a-b<0$ and stop on the part of faults with $a-b>0$ due to unconsolidated fault gouge (Kaneko and Fialko 2011). In our setup, we keep $b = 0.014$ fixed and vary the depth-dependent *a* in the inversion to perturb $a-b$. Similar to Taufiqurrahman et al. (2023), we set *a* to a constant value $a_\text {4km}$ at depths below 4 km. Between 4 km and 0 km depth, we linearly interpolate *a* from $a_\text {4km}$ towards $a_\text {0}=0.02$ at depth 0 km (Figure 9b). We use the near-fault surface displacement data at the global navigation satellite system (GNSS) stations (Melgar et al. 2020; Floyd et al. 2020) to constrain $a_\text {4km}$ in the inversion. In the parameter inference, we evaluate the posterior distribution of $a_\text {4km}$ given the uniform prior distribution in $a_\text {4km} \in [0.004,0.014]$.

**Fig. 10:**
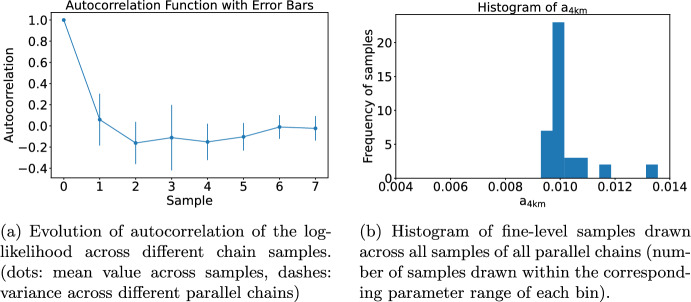
Behavior of the MLDA algorithm for the Ridecrest inversion study

We use the MLDA algorithm with 8 parallel chains, with convergence orders 3 and 4 as the two levels. We executed the workflow on the Vista supercomputer, where fused simulations with the “interleaved” strategy perform better than the “looped” strategy for the Searles Valley Earthquake scenario. We fuse $S_\text {c} = 1,2,4,8$ course-level simulations and $S_\text {f} = 1,2$ coarse-level simulations. We used a sub-sampling rate of 8, meaning that for every sample in a chain, we sample 8 coarse-level samples. Consequently, the number of order-3 runs is significantly higher than that of the order-4 runs. The resulting autocorrelation oscillates around zero after 8 samples. We plot the variation in autocorrelation across the parallel chains in Figure 10a and the histogram of the sample distribution in the parameter space $\left[ 0.004, 0.014\right]$ in Figure 10b. Because the first 3 samples for each chain showed high autocorrelation, we treat them as burn-in and discard them. Since we are inverting for a single parameter in this example workflow, we compute relatively few samples. Nevertheless, the results are in good agreement with the parallel work by Niu (2025).

**Fig. 11:**
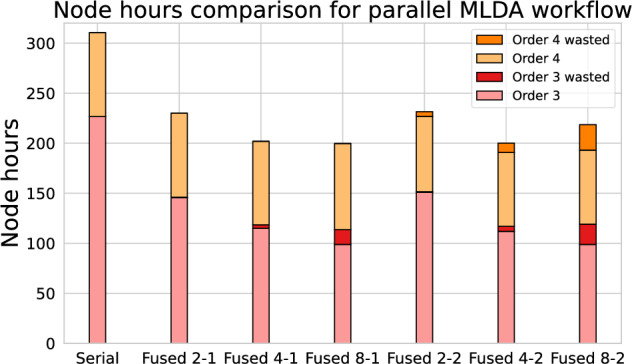
Comparison of resource usage between serial and fused simulations for the MLDA workflow. “Fused-$S_\text {c}$-$S_\text {f}$” denotes that $S_\text {c}$ simulations are fused for coarse-level simulations of the MLDA workflow, and $S_\text {f}$ simulations are fused for the fine level. Wasted simulations denote the amount of node hours wasted due to the padding of parameter batches in fused simulations

In Figure 11, we report the node hours required on Vista for different configurations of fused simulations. We used 4 nodes and ran SeisSol with 2 MPI ranks per node (one per CPU). MLDA chains were parallelized via fused simulations. The workflow with fused simulations provides a speedup of up to 1.6 (for $S_\text {c}=8$ and $S_\text {f}=1$) compared to using serial simulations for all model evaluations, reducing the required node hours by 36%. This improvement is lower than observed in Section 7, due to the interaction of the MLDA algorithm, UM-Bridge, and SeisSol. Several batches of fused simulations had to be padded with nominal values to build the prescribed ensemble size for fused simulations ($S_\text {c}$ and $S_\text {f}$). These are not used by the MLDA algorithm (and therefore “wasted”). Also, compared to the scaling studies done in Section 7.3, the MLDA workflow has a bigger variation in $a_\text {4km}$, which in turn induces bigger overhead as we have to wait for slip-rate iterations for all simulations to converge (cmp. Section 5.2).

## Conclusions

We have implemented the concept of fused ensemble simulations for complex dynamic rupture earthquake simulations with off-fault plasticity in SeisSol. In the performance-dominant wave-propagation kernel, switching from matrix to tensor data structures leads to more efficient use of SIMD instructions in the generated element-local dense-sparse and sparse-dense matrix multiplications. We also avoid padding in the dense quantities tensor, thus reducing required transfers from and to main memory. For the non-linear dynamic rupture and plasticity kernels, we implemented two strategies: a “looped” strategy that rearranges the data structure for the degree-of-freedom tensor on-the-fly to keep the implementation of kernels unchanged, and an “interleaved” strategy that avoids these memory transforms (and respective data transfers) at the cost of a slightly changed implementation of the non-linear calculations.

In our performance tests for various benchmark and production scenarios and on different CPU-based supercomputers (based on Intel and Arm CPU architectures), we observe quite diverse performance behaviour, depending on discretization order, number of fused simulations, strategy for treating non-linearities, simulation scenario (mesh properties and size), and especially CPU architecture. In the best case, a large production scenario to simulate the 2023 Kahramanmaraş earthquake, we observed a 5.54$\times$ improvement in simulations per node hour for order 3, and still a 2.93$\times$ improvement for order 4, executed on 3168 nodes (half machine) of the SuperMUC-NG supercomputer (Intel “Skylake” architecture). For a smaller benchmark scenario with TPV13, we achieved speedups of up to 4.04 on the Vista supercomputer (using the NVIDIA Grace-Grace Superchip) vs. 3.28 on the Frontera supercomputer (Intel Skylake), both for discretization order 3. Even for order 6, we see a small improvement when using the interleaved strategy: 1.2$\times$ on Frontera. For the Searles Valley earthquake scenario, as used for the MLDA workflow, we observe a maximum speedup of 2.8 for order 3, and 1.7 for order 4, both with the “interleaved” strategy and on the Vista supercomputer.

We find that the dynamic rupture and plasticity kernels, which usually have a smaller impact on performance, require careful implementation for fused simulations. At higher discretization orders, only the interleaved strategy for dynamic rupture and plasticity kernels improved performance. For the looped strategy, we think that increased memory requirements due to memory transforms cause element-local operations to fall out of cache and are thus the main reason for performance deficits. For the interleaved strategy, we also incur (smaller) overhead due to vectorization of the iterative parts of the kernels. Additional performance impacts stem from the clustered local time-stepping algorithm in SeisSol and the granularity of memory transfers.

The observed performance improvements, as well as their variability w.r.t. setup, motivate more detailed studies of the performance of the various kernels across platforms, which are beyond the scope of this paper. Further research questions include studying the impact of local time stepping, inspecting the influence of friction laws and parameters on the performance of fused simulations across different configurations. And of particular interest, of course, is the impact of fused ensemble simulations on GPU architectures. We currently fix the ensemble size to the SIMD length, but fixing it to the warp size (typically 32) on GPU architectures will likely lead to too large fused ensembles, at least for MLDA workflows. Instead, we will have to process multiple cells per warp – which is easier to accomplish on GPUs than on CPUs, due to the structure of the instruction set and the amount of registers available.

We have extended the UM-Bridge UQ and modeling interface by a batcher component to combine multiple subsequent simulation runs into fused ensembles, in a way that is transparent to the UQ algorithms. This provides the option to utilize fused ensembles for any parallel UQ algorithm and any forward solver that support UM-Bridge. We demonstrated the benefits of fused-ensemble simulations in a realistic workflow using the MLDA algorithm for Bayesian inference of a parameter governing the dynamic rupture process. As the Bayesian inference process does not always generate complete ensembles (“wasted” simulations in Figure 11), we obtain lower performance improvement compared to the scaling studies. Still, the amount of node hours consumed for this workflow is reduced by approximately 36%. Further studies are needed on more extensive workflows, such as deeper multi-level structures for MLDA or the adoption of other parallel UQ algorithms that rely on hierarchies of model resolution. For Bayesian inference problems with multiple parameters, we expect not only the total number of simulations to increase, but also the fraction of low-order simulations. For the latter, fused simulations have been shown to be most effective, and increasing the number of simulations will reduce the overhead due to incomplete ensembles. Finally, we stress that speedups from fused simulations can also be exploited in workflows for surrogate modelling or probabilistic hazard assessment, and, as with simpler parallel UQ algorithms without multiple levels, we can anticipate less overhead and speedups on par with the scaling results observed in Section 7.3.

## Data Availability

No datasets were generated or analysed during the current study.

## Footnotes

1 https://strike.scec.org/cvws/download/TPV12_13_Description_v6.pdf

2 In the following, we denote vector, matrix, and tensor variables, such as $\textbf{q}, \textbf{A},$ etc. in bold font, and use $q_i$, $A_{ij}$, etc. for the respective vector, matrix, or tensor elements.

3 PSpaMM is available at https://github.com/SeisSol/PSpaMM.

4 See detailed description by the SCEC/USGS Spontaneous Rupture Code Verification Project (https://strike.scec.org/cvws/) on https://strike.scec.org/cvws/download/TPV12_13_Description_v6.pdf.

5 The implementation is available at https://github.com/SeisSolUQ/Batcher/blob/main/batcher.py.

6 https://docs.tacc.utexas.edu/hpc/vista/

7 https://tacc.utexas.edu/systems/frontera/

8 https://doku.lrz.de/supermuc-ng-10745965.html

## Code Availability

All the files required to reproduce the simulations can be found at https://doi.org/10.5281/zenodo.18153361. SeisSol (www.seissol.org) and UM-Bridge are available as open source software. Details about installing and running SeisSol on different computers can be found at https://seissol.readthedocs.io/en/latest/build-overview.html. SeisSol is available at https://github.com/SeisSol/SeisSol/. UM-Bridge is available from https://github.com/UM-Bridge/umbridge, the batcher script can be found at https://github.com/SeisSolUQ/Batcher, and the SeisSol UMBridge server is published at https://github.com/SeisSolUQ/Seis-Bridge/tree/main/ridgecrest.
